# Effective Activation by Kynurenic Acid and Its Aminoalkylated Derivatives on M-Type K^+^ Current

**DOI:** 10.3390/ijms22031300

**Published:** 2021-01-28

**Authors:** Yi-Ching Lo, Chih-Lung Lin, Wei-Yu Fang, Bálint Lőrinczi, István Szatmári, Wan-Hsuan Chang, Ferenc Fülöp, Sheng-Nan Wu

**Affiliations:** 1 Department of Pharmacology, School of Medicine, College of Medicine, Kaohsiung Medical University, Kaohsiung 80708, Taiwan; tunafung@gmail.com (W.-Y.F.); marian14773@gmail.com (W.-H.C.); 2 Department of Medical Research, Kaohsiung Medical University Hospital, Kaohsiung 80708, Taiwan; 3 Graduate Institute of Medicine, College of Medicine, Kaohsiung Medical University, Kaohsiung 80708, Taiwan; 4 Department of Neurosurgery, Kaohsiung Medical University Hospital, Kaohsiung 80708, Taiwan; cllin@kmu.edu.tw; 5 Department of Neurosurgery, College of Medicine, Kaohsiung Medical University, Kaohsiung 80708, Taiwan; 6 Institute of Pharmaceutical Chemistry, University of Szeged, Eötvös u. 6, H-6720 Szeged, Hungary; lorinczi.balint@stud.u-szeged.hu (B.L.); szatmari.istvan@szte.hu (I.S.); fulop@pharm.u-szeged.hu (F.F.); 7 MTA-SZTE Stereochemistry Research Group, Hungarian Academy of Sciences, Eötvös u. 6, H-6720 Szeged, Hungary; 8 Institute of Basic Medical Sciences, National Cheng Kung University Medical College, Tainan City 70101, Taiwan; 9 Department of Physiology, National Cheng Kung University Medical College, Tainan City 70101, Taiwan

**Keywords:** kynurenic acid, kynurenic acid derivative, M-type K^+^ current, action potential, pituitary cell, hippocampal neuron

## Abstract

Kynurenic acid (KYNA, 4-oxoquinoline-2-carboxylic acid), an intermediate of the tryptophan metabolism, has been recognized to exert different neuroactive actions; however, the need of how it or its aminoalkylated amide derivative *N*-(2-(dimethylamino)ethyl)-3-(morpholinomethyl)-4-oxo-1,4-dihydroquinoline-2-carboxamide (KYNA-A4) exerts any effects on ion currents in excitable cells remains largely unmet. In this study, the investigations of how KYNA and other structurally similar KYNA derivatives have any adjustments on different ionic currents in pituitary GH_3_ cells and hippocampal mHippoE-14 neurons were performed by patch-clamp technique. KYNA or KYNA-A4 increased the amplitude of M-type K^+^ current (*I*_K(M)_) and concomitantly enhanced the activation time course of the current. The EC_50_ value required for KYNA- or KYNA-A4 -stimulated *I*_K(M)_ was yielded to be 18.1 or 6.4 μM, respectively. The presence of KYNA or KYNA-A4 shifted the relationship of normalized *I*_K(M)_-conductance versus membrane potential to more depolarized potential with no change in the gating charge of the current. The voltage-dependent hysteretic area of *I*_K(M)_ elicited by long-lasting triangular ramp pulse was observed in GH_3_ cells and that was increased during exposure to KYNA or KYNA-A4. In cell-attached current recordings, addition of KYNA raised the open probability of M-type K^+^ channels, along with increased mean open time of the channel. Cell exposure to KYNA or KYNA-A4 mildly inhibited delayed-rectifying K^+^ current; however, neither *erg*-mediated K^+^ current, hyperpolarization-activated cation current, nor voltage-gated Na^+^ current in GH_3_ cells was changed by KYNA or KYNA-A4. Under whole-cell, current-clamp recordings, exposure to KYNA or KYNA-A4 diminished the frequency of spontaneous action potentials; moreover, their reduction in firing frequency was attenuated by linopirdine, yet not by iberiotoxin or apamin. In hippocampal mHippoE-14 neurons, the addition of KYNA also increased the *I*_K(M)_ amplitude effectively. Taken together, the actions presented herein would be one of the noticeable mechanisms through which they modulate functional activities of excitable cells occurring in vivo.

## 1. Introduction

Kynurenic acid (KYNA) is a naturally occurring product of the normal metabolism of amino acid L-tryptophan that has been reported to inhibit NMDAR and neuronal nicotinic α_7_ receptors [1,2,3,4]. This compound, together with L-kynurenine, is thought to be an endogenous metabolite of L-tryptophan known to block *N*-methyl-D-aspartate receptor (NMDAR), and it has been frequently demonstrated to exert neuroprotective or anticonvulsant properties in the brain [2,3,5,6,7,8,9,10,11,12,13]. This compound has been indeed disclosed to inhibit NMDARs at the glycine-binding site and it can noncompetitively inhibit α_7_-nicotinic acetylcholine receptor, and through this action, it might modulate glutamate release presynaptically [1,3,14,15].

Earlier studies have demonstrated that the reduction in the astrocytic formation of KYNA could enhance glutamatergic tone in the hippocampus as well as cognitive abilities and synaptic plasticity [8]. KYNA is also thought to be a target molecule in neuroendocrinology [11]. The KYNA derivatives have been also increasingly noticed to exert various biological actions [10,16,17]. For example, as administered systemically, KYNA-A4 was previously reported to decrease population spike activity recorded from the pyramidal layer of area CA1 of the hippocampus [10]. However, the issue of how KYNA and other structurally similar compounds exert any perturbations on voltage-activated ionic currents in electrically excitable membrane is not yet thoroughly investigated, although previous experiments have reported the effectiveness of KYNA in modulating the magnitude of KCNQ gene- (KCNQ)- or HCN gene- (HCN)-encoded current [18,19].

The KCNQ2, KCNQ3, or KCNQ5 gene is recognized to encode the core subunit of K_V_7.2, K_V_7.3 or K_V_7.5 channel, respectively. The enhanced activity of these voltage-gated K^+^ channels can generate the macroscopic M-type K^+^ current (*I*_K(M)_), which is biophysically manifested by current activation in response to low-threshold voltage and, once activated, the current displays a slowly activating and deactivating property [20,21,22,23,24,25,26]. Targeting *I*_K(M)_ is noticeably viewed as an adjunctive regimen for the management of various neurological or endocrine disorders associated with neuronal over-excitability, which include cognitive dysfunction, neuropathic pain, and epilepsy [27,28,29,30]. Alternatively, previous studies have demonstrated that KYNA-mediated vasodilation and hypotension could be attributed to the activation of KCNQ channels [18,31].

**Figure 1:**
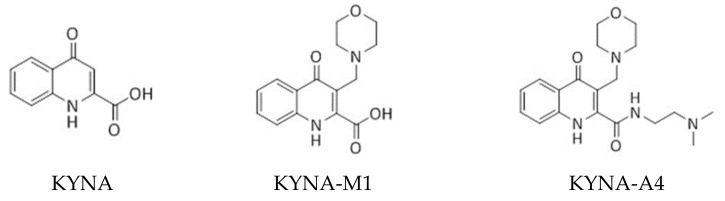
Chemical structures of kynurenic acid (KYNA, 4-hydroxyquinoline-2-carboxylic acid) and its amide derivatives (KYNA-M1, [3-(morpholinomethyl)-4-oxo-1,4-dihydroquinoline-2-carboxylic acid] and KYNA-A4, [*N*-(2-(dimethylamino)ethyl)-3-(morpholinomethyl)-4-oxo-1,4-dihydroquinoline-2-carboxamide]).

In light of the above-stated considerations, in the present study, we wanted to evaluate the possible underlying mechanisms of KYNA or its structurally similar compound KYNA-M1(3-(morpholinomethyl)-4-oxo-1,4-dihydroquinoline-2-carboxylic acid) or KYNA-A4 (*N*-(2-(dimethylamino)ethyl)-3-(morpholinomethyl)-4-oxo-1,4-dihydroquinoline-2-carboxamide) (Figure 1) on the perturbing actions on different types of voltage-gated ionic currents (e.g., *I*_K(DR)_ [delayed-rectifier K^+^ current], *I*_K(M)_, *I*_h_ [hyperpolarization-activated cation current], and *I*_Na_ [voltage-gated Na^+^ current]) in pituitary GH_3_ or R1220 cells and in hippocampal mHippoE-14 neurons.

## 2. Results

### 2.1. Effect of KYNA and KYNA Derivatives (i.e., KYNA-M1 and KYNA-A4) on M-Type K^+^ Current (I_K(M)_) in GH_3_ Cells

**Figure 2:**
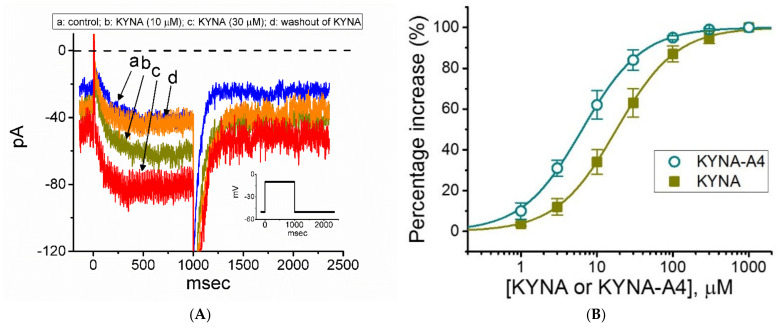
Effect of kynurenic acid (KYNA, 4-hydroxyquinoline-2-carboxylic acid) and *N*-(2-(dimethylamino)ethyl)-3-(morpholinomethyl)-4-oxo-1,4-dihydroquinoline-2-carboxamide (KYNA-A4) on M-type K^+^ current (*I*_K(M)_) recorded from GH_3_ cells. In these experiments, we kept cells to be bathed in high-K^+^, Ca^2+^-free solution, and the recording electrode was backfilled with K^+^-containing internal solution. (**A**) Representative current traces obtained in the control (a), during cell exposure to 10 μM KYNA (b) or 30 μM KYNA (c), and after washout of KYNA (d). The insert shows the voltage-clamp protocol applied, while the dashed line is zero-current level. (**B**) Concentration-dependent stimulation of KYNA (■) or KYNA-A4 (○) on the amplitude of *I*_K(M)_ (i.e., linopirdine-sensitive current) (mean ± SEM; *n* = 7). Current amplitudes during the exposure to different concentrations (1 μM to 1 mM) of KYNA or KYNA-A4 were taken at the end of the 1-s depolarizing pulse from −50 to −10 mV, and the amplitudes were then compared with those measured after subsequent addition of linopirdine (10 μM). The smooth lines with which the experimental data are overlaid were derived with the goodness-of-fitness test by the modified Hill equation detailed under Section Materials and Methods.

In the first stage, we determined whether the *I*_K(M)_ inherently in GH_3_ cells [21,25,32,33] can be subject to being modified by these compounds. As illustrated in Figure 2A,B, after 1 min of continuous exposure of cells to 10 or 30 μM KYNA caused a progressive increase in the amplitude of *I*_K(M)_ activated by long-lasting membrane depolarization. For example, KYNA at a concentration of 30 μM, *I*_K(M)_ evoked by 1-s step depolarization from −50 to −10 mV was increased to 82 ± 8 pA (*n* = 8, *p* < 0.05) from a control value of 39 ± 7 pA (*n* = 8). The value of the activation time constant of *I*_K(M)_ was concomitantly reduced from 141 ± 23 to 67 ± 12 ms (*n* = 8, *p* < 0.05). After washout of the agent, current amplitude returned to 41 ± 7 pA (*n* = 7, *p* < 0.05). Amide derivative of KYNA (KYNA-M1) also led to the raise in *I*_K(M)_ amplitude to a similar magnitude (data not shown), while KYNA-A4, another KYNA derivative was noticed to increase current amplitude more potently. Meanwhile, the *I*_K(M)_ functionally expressed in another pituitary cells (i.e., R1220 cells) was sensitive to stimulation by KYNA.

The association between the KYNA or KYNA-A4 concentration and the percentage increase in *I*_K(M)_ was further determined. Of notice, KYNA or KYNA-A4 was able to raise the amplitude of *I*_K(M)_ in a concentration-dependent manner (Figure 2B). In the presence of KYNA or KYNA-A4, the least-squares fit to the modified Hill equation, as described under Section Materials and Methods, yielded 18.1 or 6.4 μM, respectively, as the concentration required for half-maximal stimulation (i.e., EC_50_) was determined. Therefore, the emerging data reflect that the magnitude of changes in *I*_K(M)_ stimulated by KYNA-A4 tends to be higher than that by KYNA.

### 2.2. Steady-State Activation Curve of I_K(M)_ in the Absence and Presence of KYNA or KYNA-A4 in GH_3_ Cells

**Figure 3:**
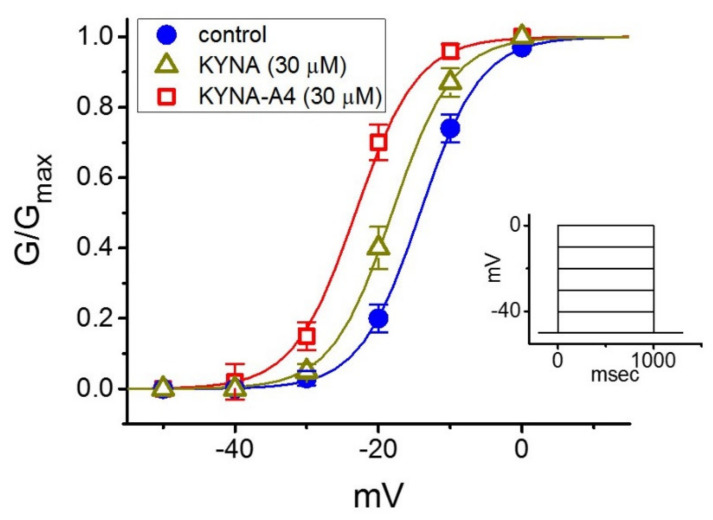
Effect of kynurenic acid (KYNA, 4-hydroxyquinoline-2-carboxylic acid) and *N*-(2-(dimethylamino)ethyl)-3-(morpholinomethyl)-4-oxo-1,4-dihydroquinoline-2-carboxamide (KYNA-A4) on the activation curve of *I*_K(M)_ in GH_3_ cells. In this set of current recordings, as the whole-cell mode was firmly established, we voltage-clamped the cell at -50 mV and a series of step commands ranging between -50 and 0 mV in 10-mV increments was applied. The relationship of conductance versus membrane potential was demonstrated in the absence (●) and presence of 30 μM KYNA (∆) or 30 μM KYNA-A4 (□) (mean ± SEM; *n* = 7). Inset shows the voltage-clamp protocol used.

We next studied whether the activation curve of *I*_K(M)_ (i.e., the *I*_K(M)_-conductance versus membrane potential) can be modified by the presence of KYNA or KYNA-A4. As illustrated in Figure 3, the relation of *I*_K(M)_ conductance versus membrane potential obtained in the control (i.e., KYNA or KYNA-A4 was not present) and during exposure to KYNA or KYNA-A4 was constructed and then analyzed. The sigmoidal curve derived from data sets was optimally fitted with the modified Boltzmann equation (described under Section Materials and Methods). That is, the values of *V*_1/2_ taken in the control, during cell exposure to 30 μM KYNA and 30 μM KYNA-A4 were −14.2 ± 1.8 mV, −18.1 ± 1.8 mV, and −23.2 ± 1.9 mV (*n* = 7), respectively, while those of the gating charge (i.e., *q*) in the control, in the presence of 30 μM KYNA and 30 μM KYNA-A4 were 5.9 ± 0.2 *e*, 6.0 ± 0.2 *e* and 6.0 ± 0.3 *e* (*n* = 7), respectively. The emerging data thus enable us to disclose that, aside from the increase in *I*_K(M)_ conductance, the presence of KYNA (30 μM) or KYNA-A4 (30 μM) was capable of causing a leftward shift along the voltage axis, despite ineffectiveness of these compounds in modifying the gating charge of the current.

### 2.3. Effect of KYNA on Voltage-Dependent Hysteresis of I_K(M)_ Elicited by Long Isosceles-Triangular Ramp Pulse

**Figure 4:**
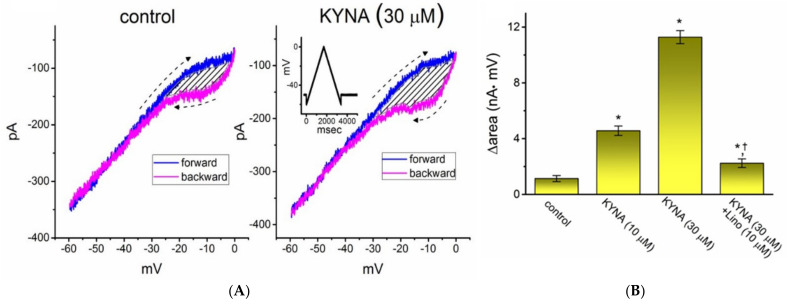
Effect of kynurenic acid (KYNA, 4-hydroxyquinoline-2-carboxylic acid) on the voltage-dependent hysteresis of *I*_K(M)_ identified from GH_3_ cells. Cells were bathed in high-K^+^, Ca^2+^-free solution, and the electrode was backfilled with K^+^-containing solution. (**A**) Representative current traces in the absence (**left**) or presence of 30 μM KYNA (**right**). Current traces were elicited in response to 3.4-s long isosceles-triangular ramp voltage command (indicated in Inset of the right panel). The dashed arrows in each panel indicate the direction of current trajectory in which time passes (—: forward; —: backward). (**B**) Summary bar graph depicting the effects of KYNA (10 or 30 μM) or KYNA (30 μM) plus linopirdine (10 μM, Lino) on the ∆area (indicated in the shaded area of (**A**)) of the voltage hysteresis (mean ± SEM; *n* = 8 for each bar). * indicates a significant difference from control (*p* < 0.05) and † indicates significant difference from the KYNA (30 μM) alone group (*p* < 0.05).

The voltage hysteresis of ionic currents (i.e., a lag in the current magnitude as the linear voltage command is changed in the opposite direction) has been demonstrated with a notable impact on the electrical signal events of different excitable cells [25,34,35,36,37]. In other words, hysteretic behavior is thought to dynamically adjust the voltage sensitivity and kinetics to optimize channel function for matching its physiological role. An urge was, therefore, made to determine how the presence of KYNA is able to adjust the strength of such hysteresis occurring in *I*_K(M)_. In this separate set of experiments, as soon as the whole-cell configuration was established, we held the examined cell in voltage clamp at −50 mV, and a long-lasting upright isosceles-triangular ramp pulse with a duration of 3.4 s (i.e., a ramp slope of ±35 mV/s) was designed and, through digital-to-analog conversion, thereafter delivered at a rate of 0.025 Hz. Of interest, as depicted in Figure 4, the trajectories of *I*_K(M)_ activated by the forward upsloping (i.e., voltage change from −60 to 0 mV) ramp pulse and by the backward downsloping (i.e., the change from 0 to −60 mV) as a function of time (as indicated by the dashed arrows in Figure 4A) were overly distinct between these two limbs. In other words, the *I*_K(M)_ amplitude elicited by the upsloping (forward) limb of the triangular voltage ramp was demonstrated to be smaller than that responding to the downsloping (backward) limb of the ramp. These observations indicate that there was a voltage-dependent hysteresis ranging between −30 and 0 mV for this type of current in these cells. The strength of the voltage hysteresis was further quantified on the basis of the difference in the area under the curvilinear trajectory in the forward (upsloping) and reverse (downsloping) direction. Figure 4B illustrates a summary of the area under the curve (i.e., ∆area indicated in the shaded area) between the forward and backward currents activated in response to a 3.4-s isosceles-triangular ramp pulse. Of note, as the whole-cell *I*_K(M)_ was established, the addition of 10 or 30 μM KYNA increased the area up to 4- or 10-fold, respectively, while the presence of 10 μM linopirdine, a well-known inhibitor of *I*_K(M)_ or KM channels [24,25], alone decreased the area by around 60%. Subsequent addition of 10 μM linopirdine, still in the presence of 30 μM KYNA, attenuated KYNA-induced increase of such hysteretic area. It is conceivable, therefore, that voltage-dependent hysteresis of *I*_K(M)_ in these cells can be enlarged by the presence of KYNA.

### 2.4. Effect of KYNA on M-Type K^+^ Channel (K_M_) Channels Recorded from GH_3_ Cells

**Figure 5:**
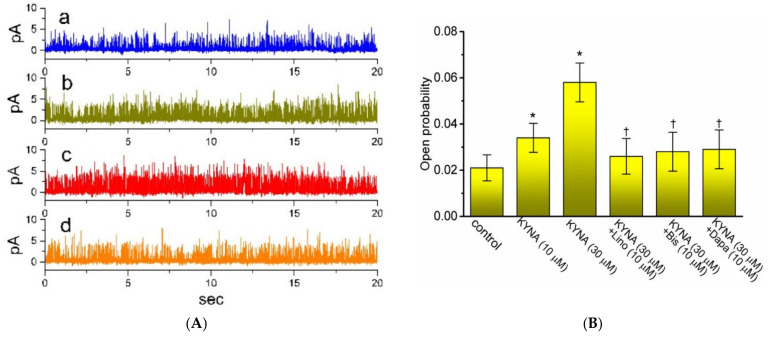
Effect of kynurenic acid (KYNA, 4-hydroxyquinoline-2-carboxylic acid) on single channel activity of M-type K^+^ (K_M_) channels identified in GH_3_ cells. In these current recordings, we bathed cells in high-K^+^, Ca^2+^-free solution and the recording electrode was backfilled with low-K^+^ (5.4 mM) solution, the composition of which is stated in Section Materials and Methods. (**A**) Original single K_M_ channels obtained in the control (a), during exposure to 10 μM KYNA (b) or 30 μM KYNA (c), and after application of 30 μM KYNA plus 10 μM linopirdine (d). The potential was held at 0 mV relative to the bath. The opening event of the channel is indicated as the upward deflection. (**B**) Summary bar graph showing effects of KYNA (10 or 30 μM), KYNA (30 μM) plus linopirdine (Lino, 10 μM), KYNA (30 μM) plus bisoprolol (Bis, 10 μM) and KYNA (30 μM) plus dapagliflorizin (Dapa, 10 μM) on the channel opening probability of K_M_ channels recorded from GH_3_ cells (mean ± SEM; *n* = 7 for each bar). Channel activity was measured at 0 mV relative to the bath. * indicates a significant difference from control (i.e., KYNA was not present) (*p* < 0.05), while † indicates significant difference from KYNA (30 μM) alone group (*p* < 0.05).

The KYNA-stimulated whole-cell *I*_K(M)_ observed above could arise from changes in either channel open probability, unitary amplitude, or gating kinetics of the K_M_ channel. The reasons therefore urged us to examine the single-channel recording of the channel. In these cell-attached current recordings, we suspended cells in high-K^+^, Ca^2+^-free solution, and the recording electrode was filled with low-K^+^ (5.4 mM) solution. As illustrated in Figure 5, when the examined cell was maintained at 0 mV, the activity of single-K_M_ channel was robustly detected [32]. As KYNA was applied to the bath, the channel open probability was progressively raised. For example, the presence of 30 μM KYNA significantly increased the channel open probability from 0.021 ± 0.006 to 0.058 ± 0.008 (*n* = 8, *p* < 0.05); however, minimal change in the single-channel amplitude was demonstrated. The mean open time of K_M_ channels was concurrently lengthened to 6.1 ± 0.3 ms (*n* = 7, *p* < 0.05) from a control value of 3.2 ± 0.2 ms (*n* = 7). Moreover, in the continued presence of KYNA, further application of either linopirdine, bisoprolol, or dapagliflorizin was able to decrease KYNA-enhanced probability of K_M_-channel openings significantly. Bisoprolol and dapagliflorizin were previously reported to inhibit *I*_K(M)_ amplitude [32,38].

### 2.5. Effect KYNA on Delayed-Rectified K^+^ Current (I_K(DR)_) in Pituitary Tumor (GH_3_) Cells

**Figure 6:**
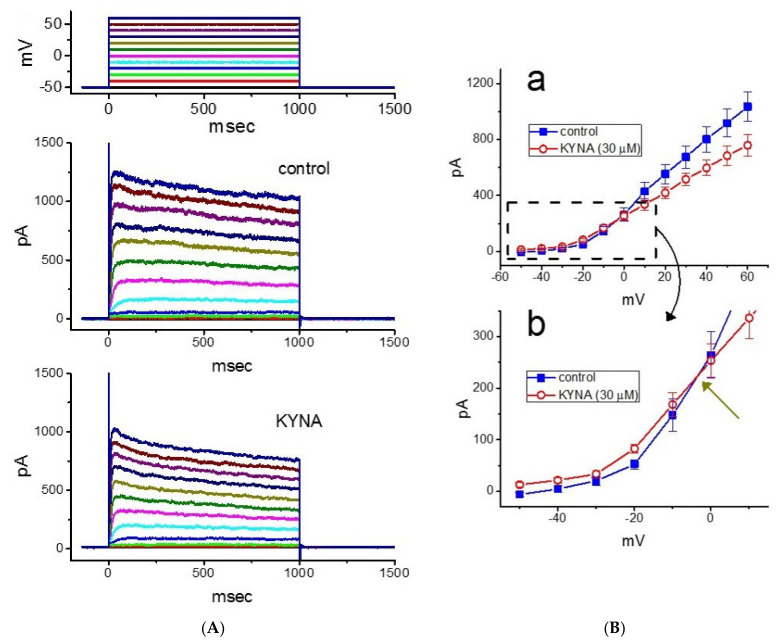
Effect of kynurenic acid (KYNA, 4-hydroxyquinoline-2-carboxylic acid) on delayed-rectifier K^+^ current (*I*_K(DR)_) in GH_3_ cells. The experiments were undertaken in cells bathed in Ca^2+^-free, Tyrode’s solution, and we filled up the electrode with K^+^-containing solution. (**A**) Representative current traces obtained in the control (upper) and during cell exposure to 30 μM KYNA. The voltage-clamp profile was demonstrated in the uppermost part. (**Ba**) Mean *I-V* relations of *I*_K(DR)_ taken in the absence (■) and presence (○) of 30 μM KYNA (mean ± SEM; *n* = 8 for each point). Current amplitude was taken at each depolarizing pulse from a holding potential of −50 mV. Panel (**Bb**) shows an expanded graph from (**Ba**), while the arrow indicates a crossover point between these two *I-V* curves.

In the next stage of the experiments, we evaluated possible perturbations of KYNA on *I*_K(DR)_ in these cells. To record *I*_K(DR)_, cells were bathed in Ca^2+^-free Tyrode’s solution which contained tetrodotoxin (TTX, 1 μM) and CdCl_2_ (0.5 mM), and the recording pipette was filled up with K^+^-containing solution. Tetrodotoxin (TTX) or CdCl_2_ was used to block Na^+^ or Ca^2+^ currents, respectively, while Ca^2+^-free Tyrode’s solution used as bathing solution was to avoid any contamination of Ca^2+^-activated K^+^ currents [25,39]. When the examined cell was maintained in voltage clamp at −50 mV and a series of voltage pulses ranging from −50 to +60 mV in 10-mV increments with a duration of 1 s was applied, a family of the outwardly directed K^+^ currents was elicited (Figure 6A,B). These outward currents, which displayed an outwardly rectifying property with fast activation and deactivation kinetics, were sensitive to block by tetraethylammonium (TEA), but not by iberiotoxin or apamin, and was identified as *I*_K(DR)_ [39]. Iberiotoxin or apamin is the blocker of the large- or small-conductance Ca^2+^-activated K^+^ channels, respectively. When cells were exposed to KYNA (30 μM), the amplitude of the outwardly directed *I*_K(DR)_ in response to membrane depolarization was reduced with little or no concurrent change in current inactivation. For example, when the examined cells were depolarized from −50 to +50 mV, KYNA at a concentration of 10 μM decreased the current amplitude by 26 ± 3 % from 917 ± 99 to 683 ± 68 pA (*n* = 12). After washout of the agent, current amplitude was returned to 892 ± 93 pA (*n* = 11). The current-voltage (*I-V*) relationship of *I*_K(DR)_ with or without addition of KYNA was constructed and is then illustrated in Figure 6B, indicating that the presence of this compound mildly inhibited the amplitude of *I*_K(DR)_. Additionally, it was noticed that the current amplitudes at the voltages ranging between −50 and −10 mV were increased in the presence of KYNA. Figure 6Bb depicts an *I-V* relationship of KYNA-sensitive current (i.e., difference between current amplitude obtained in the absence and presence of 30 μM KYNA). The enhanced current amplitude obtained at the voltage below 0 mV was attenuated by further addition of linopirdine (10 μM), reflecting that, under these experimental conditions, the component appears to belong to *I*_K(M)_ that is stimulated by KYNA.

### 2.6. Lack of KYNA on Erg-Mediated K^+^ Current (I_K(erg)_)

**Figure 7:**
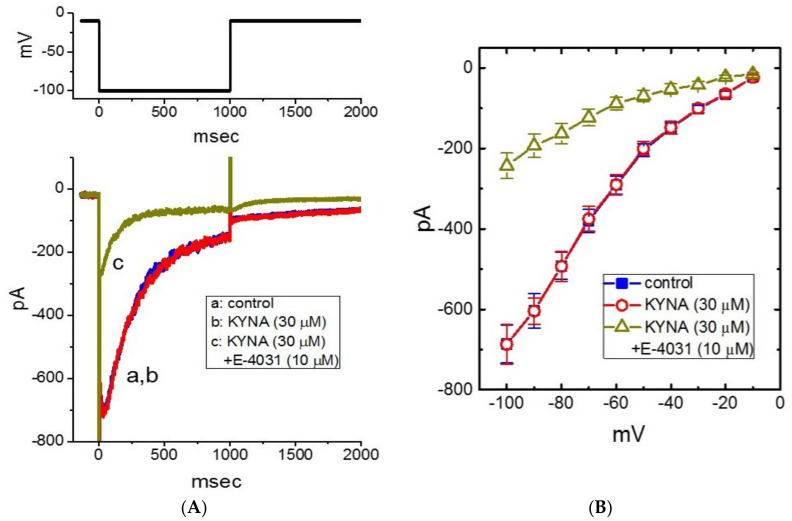
Lack of kynurenic acid (KYNA, 4-hydroxyquinoline-2-carboxylic acid) effect of *erg*-mediated K^+^ current (*I*_K(erg)_). In this set of experiments, we kept cells in high-K^+^, Ca^2+^-free solution and the electrode was backfilled with K^+^-containing solution. (**A**) Representative current traces (i.e., the inwardly directed *I*_K(erg)_) obtained in the control (a) and during exposure to 30 μM KYNA (b) or 30 μM KYNA plus 10 μM E-4031 (c). The upper part shows the voltage-clamp protocol used to evoke *I*_K(erg)_. (**B**) Mean *I-V* relationships of *I*_K(erg)_ obtained in the absence (■) of 30 μM KYNA (○) or 30 μM KYNA plus 10 μM E-4031 (∆) (mean ± SEM; *n* = 7 for each point). Current amplitude was measured at the beginning of each hyperpolarizing pulse. Of notice, the overall *I-V* relationship of *I*_K(erg)_ between the absence and presence of KYNA (30 μM) did not differ.

In the next set of experiments, we examined whether KYNA could perturb different types of voltage-gated K^+^ current (e.g., *I*_K(erg)_). In these current recordings, we kept cells in high-K^+^, Ca^2+^-free solution, and the cell was held in voltage clamp at −10 mV and the 1-s hyperpolarizing pulse to −100 mV was applied to evoke the inwardly directed *I*_K(erg)_, which is sensitive to block by thyrotropin releasing hormone (1 μM) [40]. As illustrated in Figure 7A,B, the application of KYNA (30 μM) resulted in no change in *I*_K(erg)_ amplitude. The mean *I-V* relationship of *I*_K(erg)_ amplitude measured at different levels of 1-s hyperpolarizing pulses taken with or without KYNA (30 μM) addition was depicted in Figure 7B. For example, as the cell was 1-s depolarized from -10 to -100 mV, *I*_K(erg)_ obtained in the control (i.e., KYNA was not present) was measured to be 686 ± 47 pA (*n* = 8), a value that did not differ significantly from that in the presence of 30 μM KYNA (687 ± 49 pA, *n* = 8, *p* > 0.05). Moreover, as cells were continually exposed to 30 μM KYNA, subsequent addition of E-4031 (10 μM) resulted in a significant reduction of deactivating *I*_K(erg)_ in response to membrane hyperpolarization (Figure 7B). Therefore, unlike *I*_K(M)_ or *I*_K(DR)_, the *I*_K(erg)_ in GH_3_ cells is resistant to any adjustments by KYNA.

### 2.7. Inability of KYNA to Modify Hyperpolarization-Activated Cation Current (I_h_)

**Figure 8:**
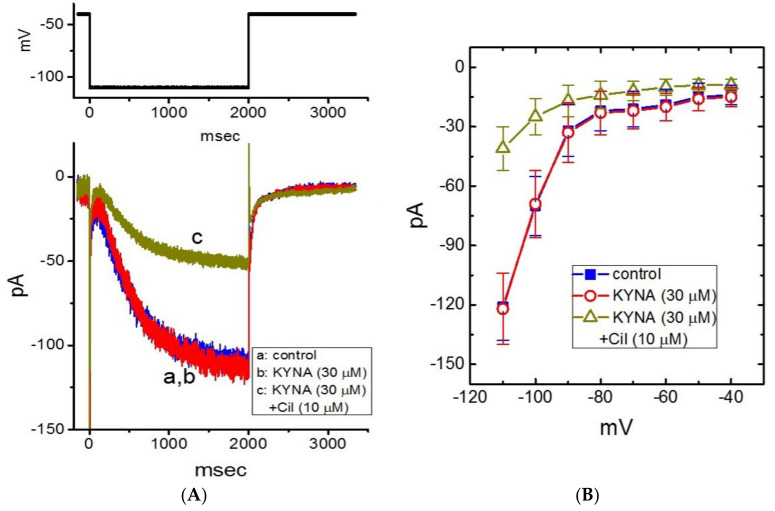
Lack of kynurenic acid (KYNA, 4-hydroxyquinoline-2-carboxylic acid) effect on the hyperpolarization-activated cation current (*I*_h_) recorded from GH_3_ cells. The whole-cell current recordings were conducted in cells bathed in Ca^2+^-free, Tyrode’s solution containing 1 μM TTX and 0.5 mM CdCl_2_, and the recording electrode was filled up with K^+^-containing solution. (**A**) Representative current traces obtained in the control (a, KYNA was not present), and during exposure to KYNA (30 μM) (b) or to KYNA (30 μM) plus cilobradine (Cil, 10 μM) (c). The upper part is the voltage-clamp protocol delivered. (**B**) Mean *I-V* relationship of *I*_h_ taken in the absence (■) and presence of KYNA (30 μM) (○) or KYNA (30 μM) plus cilobradine (Cil, 10 μM) (∆) (mean ± SEM; *n* = 8 for each point). Current amplitude was taken at the end of 2-s hyperpolarizing pulse to a series of voltages ranging between −110 and −40 mV from a holding potential of −40 mV. Of notice, there is void of KYNA effect on the amplitude or time course of *I*_h_ evoked throughout the entire voltage-clamp steps examined; however, further addition of cilobradine, still in the presence of KYNA, can decrease *I*_h_ amplitude effectively.

Previous work has demonstrated the ability of KYNA to decrease the amplitude of *I*_h_ or heart rate [15,19,41]. For these reasons, we further investigated whether the presence of KYNA could perturb the amplitude and gating of *I*_h_ in GH_3_ cells [42]. In these experiments, cells were bathed in Ca^2+^-free, Tyrode’s solution and we filled up the electrode by using K^+^-containing solution. When the whole-cell configuration was achieved, we held the cell in voltage clamp at −40 mV and a 2-s hyperpolarizing pulse was applied to evoke the inwardly directed *I*_h_ with a slowly activating time course (Figure 8A,B). As GH_3_ cells were exposed to 30 μM KYNA, the *I*_h_ amplitude at the level of −110 mV was not changed (121 ± 17 pA (in the control) versus 122 ± 18 pA (in the presence of 30 μM KYNA); *n* = 8, *p* > 0.05). However, in the continued presence of 30 μM KYNA, further addition of 10 μM cilobradine was able to decrease *I*_h_ amplitude to 41 ± 11 pA (*n* = 8, *p* < 0.05). Cilobradine was previously reported to block *I*_h_ [43]. Mean *I-V* relationship of *I*_h_ amplitude obtained in the absence and presence of KYNA or KYNA plus 10 μM cilobradine is illustrated in Figure 8B. It is therefore reasonable to assume that the *I*_h_ in GH_3_ cells tends to be resistant to KYNA.

### 2.8. Ineffectiveness of KYNA in Modifying Voltage-Gated Na^+^ Current (I_Na_) in GH_3_ Cells

**Figure 9:**
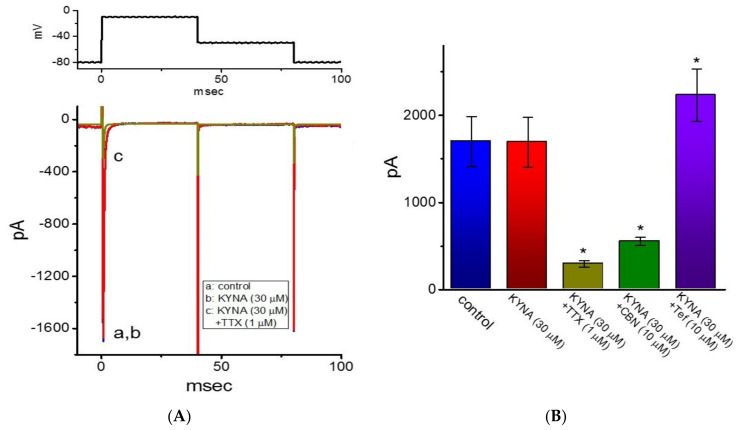
Inability of kynurenic acid (KYNA, 4-hydroxyquinoline-2-carboxylic acid) to perturb the amplitude or gating of voltage-gated Na^+^ current (*I*_Na_) in GH_3_ cells. In these whole-cell experiments, cells were kept to be immersed in Ca^2+^-free, Tyrode’s solution containing 10 mM TEA and 0.5 mM CdCl_2_, while we filled up the electrode with Cs^+^-containing internal solution. (**A**) Representative *I*_Na_ traces taken in the control (i.e., KYNA was not present, a) and during cell exposure to 30 μM KYNA (b) or 30 μM KYNA plus 1 μM TTX (c). The voltage-clamp protocol used to elicit *I*_Na_ is depicted in the upper part. (**B**) Summary bar graph showing effects of KYNA, KYNA plus TTX, KYNA plus columbianadin (CBN, 10 μM), or KYNA plus tefluthrin (Tef, 10 μM). Each bar represents the mean ± SEM (*n* = 7). * indicates a significant difference from KYNA (30 μM) alone group (*p* < 0.05).

Riluzole is recognized to be an antagonist of NMDA receptors and beneficial for the management of amyotrophic lateral sclerosis [6]. It was previously demonstrated to inhibit *I*_Na_ effectively in skeletal muscle cells [44]. For these reasons, we further extended to explore whether KYNA is able to produce any effects on *I*_Na_. In this set of current recordings, we bathed cells in Ca^2+^-free, Tyrode’s solution containing 10 mM TEA, and the recording electrode was backfilled with Cs^+^-containing solution. As the whole-cell mode was achieved, we voltage-clamped the cell at a holding potential of −80 mV and the rapid depolarizing pulse to −10 mV was applied to evoke *I*_Na_ in these cells. As illustrated in Figure 9, cell exposure to 30 μM KYNA produced minimal changes in the amplitude of *I*_Na_. However, the inability of 30 μM KYNA to decrease peak amplitude of *I*_Na_ was demonstrated (1.7 ± 0.3 nA (in the control) versus 1.7 ± 0.3 nA (in the presence of 30 μM KYNA); *n* = 7, *p* > 0.05). Moreover, as cells were continually exposed to 30 μM KYNA, a further addition of TTX (1 μM) or columbianadin (10 μM) decreased peak *I*_Na_ amplitude to 0.30 ± 0.04 nA (*n* = 7, *p* < 0.05) or 0.55 ± 0.05 nA (*n* = 7, *p* < 0.05), respectively; however, that of tefluthrin raised peak *I*_Na_ to 2.2 ± 0.30 nA (*n* = 7, *p* < 0.05). Columbianadin was a bioactive coumarin-type compound reported to inhibit *I*_Na_ [45], while tefluthrin was previously reported to activate *I*_Na_ [46]. As such, the presence of KYNA was unable to modify the amplitude and gating of *I*_Na_ identified in GH_3_ cells.

### 2.9. Effect of KYNA or KYNA-A4 on Spontaneous Action Potentials (APs) Recorded from GH_3_ Cells

**Figure 10:**
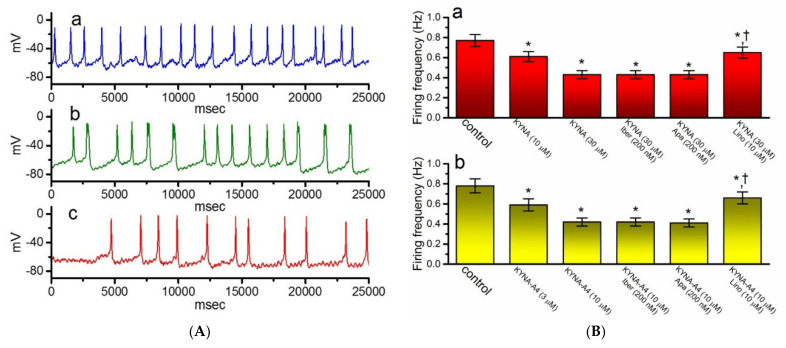
Effect of kynurenic acid (KYNA, 4-hydroxyquinoline-2-carboxylic acid) and *N*-(2-(dimethylamino)ethyl)-3-(morpholinomethyl)-4-oxo-1,4-dihydroquinoline-2-carboxamide (KYNA-A4) on spontaneous action potentials (APs) recorded from GH_3_ cells. In this set of the experiments, cells were bathed in normal Tyrode’s solution, the electrode was filled with K^+^-containing solution, and the whole-cell current-clamp voltage recordings were performed. (**A**) Original potential traces obtained in the control (a) and during exposure to 10 μM KYNA (b) or 30 μM KYNA (c). In (**B**), (**Ba**) illustrates summary bar graph depicting effects of KYNA (10 or 30 μM), KYNA (30 μM) plus iberiotoxin (Iber, 200 nM), KYNA (30 μM) plus apamin (Apa, 200 nM), and KYNA (30 μM) plus linopirdine (Lino, 10 μM) on the frequency of spontaneous APs in GH_3_ cells, while (**Bb**) shows those of KYNA-A4 (3 or 10 μM), KYNA-A4 (10 μM) plus Iber (200 nM), KYNA-A4 (10 μM) plus Apa (200 nM), and KYNA-A4 (10 μM) plus Lino (10 μM) on firing frequency. Each bar indicates the mean ± SEM (*n* = 7). * indicates a significant difference from controls (*p* < 0.05) and † indicates significant difference from KYNA (30 μM) or KYNA-A4 (10 μM) alone group (*p* < 0.05).

In another separate series of experiments, we wanted to explore whether the presence of KYNA or KYNA-A4 and the related compounds perturb any changes in membrane potential in these cells. We kept the examined cells to be bathed in normal Tyrode’s solution containing 1.8 mM CaCl_2_ and the recording pipette was filled with K^+^-containing solution. As illustrated in Figure 10A,B, when the current-clamp voltage recordings were achieved, spontaneous APs with a firing frequency of 0.77 ± 0.06 Hz (*n* = 7) were robustly detected. Of notice, as GH_3_ cells were exposed to KYNA, the cell membrane became progressively hyperpolarized and the firing frequency was concurrently decreased, as demonstrated by a significant reduction in the firing frequency to 0.42 ± 0.04 Hz (*n* = 7, *p* < 0.05) during exposure to 30 μM KYNA. In the continued presence of 30 μM KYNA, further application of neither iberiotoxin nor apamin had any effects on spontaneous APs; however, that of 10 μM linopirdine did significantly reverse KYNA-mediated decrease of firing frequency to 0.65 ± 0.06 Hz (*n* = 7, *p* < 0.05). Similar results were also found in the presence of KYNA-A4 (3 or 10 μM) (Figure 10Bb). It is therefore conceivable from these results that the observed effect of KYNA or KYNA-A4 on the firing patterns of GH_3_ cells could be explained by its stimulation of *I*_K(M)_.

### 2.10. Effect of KYNA and KYNA Plus Linopirdine on I_K(M)_ Measured from Hippocampal mHippoE-14 Neurons

**Figure 11:**
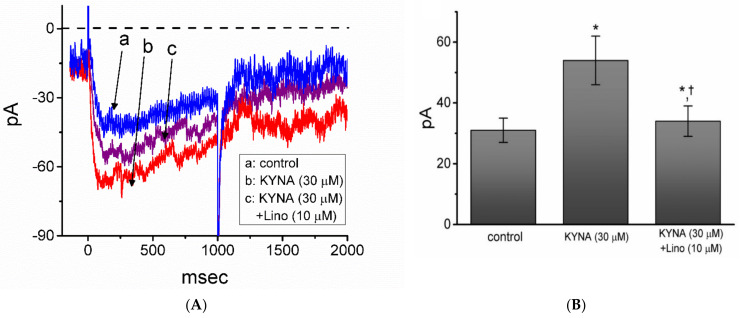
Effect of kynurenic acid (KYNA, 4-hydroxyquinoline-2-carboxylic acid) on *I*_K(M)_ identified from hippocampal mHippoE-14 neurons. For this set of current recordings, we kept cells immersed in high-K^+^, Ca^2+^-free solution containing 1 μM TTX, and we thereafter backfilled the electrode with K^+^-containing internal solution. (**A**) Representative current traces activated by membrane depolarization from -50 to -10 mV with a duration of 1 s. The dashed line shows zero-current level. a: control; b: 30 μM KYNA; c: 30 μM KYNA plus 10 μM linopirdine (Lino). (**B**) Summary bar graph showing effect of KYNA (30 μM) or KYNA (30 μM) plus linopirdine (Lino, 10 μM) on the *I*_K(M)_ amplitude recorded from mHippoE-14 neurons (mean ± SEM; *n* = 7 for each bar). Current amplitudes taken in the absence and presence of KYNA or KYNA plus linopirdine were measured at the end of 1-s depolarizing pulse from −50 to −10 mV. * indicates a significant difference from control (*p* < 0.05) and † indicates significant difference from KYNA (30 μM) alone group (*p* < 0.05).

In a final stage of experiments, we further tested whether the *I*_K(M)_ present in different types of excitable cells (e.g., hippocampal mHippoE-14 neurons) is sensitive to adjustments by KYNA. Cells were bathed in high-K^+^, Ca^2+^-free solution and the electrode used was filled with K^+^-containing internal solution. As whole-cell configuration was established, we maintained the examined cell in voltage clamp at -50 mV and the depolarizing pulse to -10 mV with a duration was applied to evoke *I*_K(M)_ [47,48]. The high-K^+^, Ca^2+^-free solution used as the extracellular solution in these experiments is to minimize the contamination of Ca^2+^-activated K^+^ channels in these cells [47]. As illustrated in Figure 11, as mHippoE-14 cells were exposed to 30 μM KYNA, the *I*_K(M)_ amplitude was increased to 54 ± 8 pA (*n* = 7, *p* < 0.05) from a control value of 31 ± 4 pA (*n* = 7). In the continued presence of 30 μM KYNA, subsequent addition of linopirdine (10 μM) was effective in attenuating KYNA-stimulated *I*_K(M)_ in these cells, as demonstrated by a significant reduction of current amplitude to 34 ± 5 pA (*n* = 7, *p* < 0.05). Meanwhile, the presence of 30 μM KYNA-A4 increased *I*_K(M)_ amplitude from 31 ± 4 to 72 ± 9 pA (*n* = 7, *p* < 0.05). Therefore, similar to the experimental results described in GH_3_ cells, *I*_K(M)_ amplitude in response to membrane depolarization was sensitive to simulation by KYNA in mHippoE-14 neurons.

## 3. Discussion

In this study, we provide the evidence to disclose that in pituitary GH_3_ cells, KYNA or KYNA-A4 produces a stimulatory effect on *I*_K(M)_ in a concentration-, voltage-, and state-dependent fashion. On the basis of the concentration-dependent relationship (i.e., the modified Hill equation), the value of EC_50_ required for KYNA- or KYNA-A4-mediated stimulation of *I*_K(M)_ identified from GH_3_ cells were calculated to be 18.1 or 6.4 μM, respectively. The EC_50_ value of KYNA-stimulated *I*_K(M)_ appeared to be lower than that for its inhibition of NR1a/NR2A receptors or AMPA-evoked currents [7]. The relationship of *I*_K(M)_-conductance versus membrane potential taken in the presence of KYNA (30 μM) or KYNA-A4 (30 μM) was noticed to produce a leftward shift along the voltage axis by about 4 or 9 mV, respectively. As such, there is anticipated to be a pertinent link between its effects on endocrine or neuroendocrine cells and the stimulatory effect on *I*_K(M)_. However, whether such differential action by KYNA and KYNA-A4 on *I*_K(M)_ could occur the in vivo studies needs to be further resolved.

In addition to the increased *I*_K(M)_ amplitude, KYNA or KYNA-A4 was able to shorten the activation time constant of the current. Stimulation of *I*_K(M)_ caused by KYNA thus does not become instantaneous, yet it develops with time once the K_M_-channels are opened upon membrane depolarization, thereby leading to an apparent increase in current activation. In keeping with these observations, single-channel current recordings were found to prolong mean open time of K_M_ channels in the presence of KYNA. In this scenario, the increase in both probability openings and mean open times of K_M_ channels produced by KYNA or its amide derivatives would be mainly responsible for its increase of macroscopic *I*_K(M)_ amplitude carried through these channels, despite their ineffectiveness in changing the single-channel amplitude. In this regard, KYNA or its structurally similar compounds (e.g., KYNA-A4) would be expected to be valuable tools for probing the structure and function of K_M_ channels [16,17,49].

In accordance with previous observations [25], the voltage-dependent hysteresis of *I*_K(M)_ in response to the long isosceles-triangular ramp pulse was demonstrated in GH_3_ cells. The strength of such hysteretic perturbations was recently described to serve a role in tuning the activity of K_M_ channels to respond as they are needed [37]. We also further determined the possible adjustments of KYNA on such non-equilibrium property of *I*_K(M)_ present in GH_3_ cells. The data reflected that the presence of KYNA allowed for an increase in the hysteretic strength efficiently (i.e., ∆area indicated in the shaded area of Figure 4A) linked to the voltage-dependent activation of *I*_K(M)_. Therefore, it is possible that intrinsic changes in the voltage dependence of the voltage sensing machinery of K_M_ channels (i.e., voltage-sensing domain relaxation) would be dynamically adjusted during exposure to KYNA or its derivatives [25,35].

Distinguishable to some extent from previous studies reflecting the effectiveness of KYNA in decreasing the amplitude of *I*_h_ or heart rate [15,19,41], in the present study, we were unable to observe any adjustments of KYNA or KYNA-A4 on the amplitude and gating of *I*_h_ activated by long-lasting membrane hyperpolarization. However, as GH_3_ cells were continually exposed to KYNA, a further addition of ivabradine effectively suppressed the amplitude of *I*_h_. Ivabradine was known to be an inhibitor of HCN-encoded current [50]. It is therefore possible that KYNA-induced analgesia tends to be indirectly associated with its changes in *I*_h_ amplitude and that it could be better explained by its activation of *I*_K(M)_. Moreover, unlike the effect of flupirtine, known to be an activator of *I*_K(M)_, on *I*_K(DR)_ described previously in motoneuron-like cells [51], the inactivation kinetics of *I*_K(DR)_ remained unchanged during exposure to KYNA or KYNA-A4, although flupirtine could inhibit the amplitude of *I*_K(DR)_.

Because of its polar structure, KYNA is thought to have poor CNS penetration. However, its precursor kynurenine, which easily crosses the blood-brain-barrier, is rapidly transported into astrocytes upon entry into the brain. Once formed in the astrocytes, KYNA can be readily released into the extracellular milieu [2]. Alternatively, recent studies have shown that the synthetic amide derivatives of KYNA (e.g., KYNA-A4) investigated in this study could readily cross the barrier [10,16,49]. In our study, the addition of KYNA was noted to activate *I*_K(M)_ effectively in hippocampal mHippoE-14 neurons. It is therefore reasonable to propose that these derivatives through their stimulation of *I*_K(M)_ could be beneficial for the treatment of different psychiatric or neurological disorders [2,5,8,11].

KYNA has been reported to be an antagonist of NMDA receptor [12]. Earlier work has reflected that addition of glutamate could depolarize pituitary cells and facilitate Ca^2+^ influx from the exterior [52,53]. Glutamate-mediated Ca^2+^ rise was also demonstrated to be attenuated by further addition of KYNA [53]. However, L-glutamate (1 mM) alone did not affect *I*_K(M)_ amplitude in GH_3_ cells; moreover, as L-glutamate (1 mM) was continually present, the subsequent addition of KYNA or KYNA-A4 still effectively increased *I*_K(M)_ in GH_3_ cells. We also observed that KYNA or KYNA-A4 depressed the firing frequency of spontaneous APs, thereby leading to the reduction of cytosolic Ca^2+^. Therefore, it is possible that the stimulatory effect of these agents on *I*_K(M)_ seen in GH_3_ cells is attributed to be in great part the result of an interaction with the K_M_ channel. Whether KYNA- or KYNA-A4-stimulated *I*_K(M)_ is mediated through the elicitation of G protein(s) (e.g., G-protein-coupled orphan receptor (GPR35)) [9,19,54,55] remains to be resolved, since it was previously reported that the voltage-dependent activity of *I*_K(M)_ could be modulated by phosphatidylinositol 4,5-bisphosphate (PIP_2_) [20,56]. Alternatively, the functional expression of GPR35 has not been thus far reported in pituitary lactotrophs.

KYNA-A4 is more effective in stimulating *I*_K(M)_ than KYNA or KYNA-M1, although none of them could perturb *I*_K(erg)_, *I*_h_ or *I*_Na_. It therefore appears that in the KYNA molecule, the tertiary nitrogen containing amide side-chain (*N,N*-dimethylaminoethyl-amide group at position 2) could enhance the activation of *I*_K(M)_ in GH_3_ cells, possibly owing to the less polar properties of KYNA-A4 as compared with those in KYNA. Nonetheless, the decreased firing of APs caused by these compounds detected in GH_3_ cells could be attributed to the stimulation of *I*_K(M)_ [32]. However, it needs to be mentioned that the inhibition by these compounds of *I*_K(DR)_ in these cells might partly contribute to their effectiveness in their perturbations on spontaneous APs with high frequency, since it can lessen the strength of resurgent K^+^ currents [57]. Of also note, awareness needs to be strengthened in attributing its aberrant use to the antagonistic effect on the NMDAR activity [8,12,14,15,53,58].

Previous studies have demonstrated that kynurenine, thought to be an endothelium-derived relaxing factor, can relax blood vessel possibly through the activation of adenylate cyclase [59]. However, in this study, in the continued presence of either KYNA or KYNA-M1, subsequent application of 2′-5′-dideoxyadenosine (10 μM), a cell-permeable inhibitor of adenylate cyclase, did not attenuate its stimulation of *I*_K(M)_ in GH_3_ cells, although further application of thyrotropin releasing hormone (1 μM) did reverse it. Therefore, it seems unlikely that the activation of *I*_K(M)_ caused by these two agents is mediated through their adjustments in the activity of adenylate cyclase.

It should also be mentioned that in some of electrophysiological recordings, KYNA (i.e., around 300 μM) had been put into the bathing solution for tissues or cells studied, in attempts to preclude any possible interference with excitatory glutaminergic neurotransmission appearing in the preparations [60,61,62,63,64,65,66]. Under this scenario, the experiments results could have been ambiguous (i.e., either under- or over-emphasized) owing possibly to the direct actions on ionic currents (e.g., the overstimulation of *I*_K(M)_) presented herein.

In light of the present findings, the perturbations by KYNA or KYNA-A4 on the amplitude and gating of *I*_K(M)_ provide an insight through which they modulate functional activities in excitable cells.

## 4. Materials and Methods 

### 4.1. Drugs, Chemicals, and Solutions Used in This Study

Kynurenic acid (KYNA, 4-oxoquinoline-2-carboxylic acid, C_10_H_7_NO_3_), 2′,5′-dideoxyadenoine, cilobradine (Cil, DK-AH269), L-glutamate, linopirdine, ODQ (1*H*-[1,2,4]oxadiazolol-[4,3a] quinoxalin-1-one), tefluthrin (Tef) tetraethylammonium chloride (TEA), tetrodotoxin (TTX), and thyrotropin releasing hormone were acquired from Sigma-Aldrich (Merck, Taipei, Taiwan), while iberiotoxin and apamin were obtained from Alomone (Yu Shing Bio-Tech, Taipei, Taiwan), E-4031 was from Enzo (Blossom Biotechnologies, Taipei, Taiwan), bisoprolol was from Tocris (Union Biomed, Taipei, Taiwan), and columbianadin (CBN) and dapagliflozin (DAPA) were from Cayman (Asia Bioscience, Taipei, Taiwan).

KYNA amide derivative (e.g., 3-(morpholinomethyl)-4-oxo-1,4-dihydroquinoline-2-carboxylic acid, C_15_H_16_N_2_O_4_, [KYNA-M1], and *N*-(2-(dimethylamino)ethyl)-3-(morpholinomethyl)-4-oxo-1,4-dihydroquinoline-2-carboxamide, C_19_H_26_N_4_O_3_, [KYNA-A4] were synthesized at Institute of Pharmaceutical Chemistry, University of Szeged, Szeged, Hungary according to literature methods [17]. The chemical structures of KYNA, KYNA-M1, and KYNA-A4 are illustrated in Figure 1. Culture media (e.g., Ham’s F-12 medium), horse or fetal calf serum, L-glutamine, and trypsin/EDTA were acquired from HyClone^TM^ (Thermo Fisher; Level Biotech, Tainan, Taiwan), whereas other chemicals, including CdCl_2_, CsCl, CsOH, aspartic acid, and HEPES, were of the best available quality, mostly at analytical grade.

The composition of extracellular solution (i.e., HEPES-buffered normal Tyrode’s solution) was as follows (in mM): NaCl 136.5, KCl 5.4, CaCl_2_ 1.8, MgCl_2_ 0.53, glucose 5.5, and HEPES-NaOH buffer 5 (pH 7.4). To record either membrane potential or the current through *I*_K(M)_, *I*_K(erg)_ or *I*_h_, the recording electrodes used were backfilled with the following solution (in mM): K-aspartate 130, KCl 20, KH_2_PO_4_ 1, MgCl_2_ 1, EGTA 0.1, Na_2_ATP 3, Na_2_GTP 0.1, and HEPES-KOH buffer 5 (pH 7.2). To record current through *I*_K(M)_ or *I*_K(erg)_, we used high K^+^-bathing solution containing the following (in mM): KCl 145, MgCl_2_ 0.53, and HEPES-KOH buffer 5 (pH 7.4). To measure the activity of single K_M_ channels, the pipette solution was composed of the following (in mM): NaCl 136.5, KCl 5.4, MgCl_2_ 0.53, and HEPES-NaOH buffer 5 (pH 7.4). All solutions were prepared using deionized water from a Milli-Q water purification system (Merck Millipore, Taipei, Taiwan). The pipette solution and culture media were filtered on the day of use with Acrodisc^®^ syringe filter with Supor^®^ membrane (0.22 μm in pore size) (Bio-Check, New Taipei City, Taiwan). 

### 4.2. Cell Preparations

GH_3_ pituitary tumor cells, acquired from the Bioresources Collection and Research Center ((BCRC, catalog number: 60015); Hsinchu, Taiwan; originally derived from the American Type Culture Collection (ATCC^®^ (CCL-82.1^TM^, catalog number: 82.1)), were cultured in Ham’s F-12 medium supplemented with 2.5% fetal calf serum (*v/v* percent), 15% horse serum (*v/v* percent), and 2 mM L-glutamine at 37 °C in a humidified environment of 5% CO_2_/95% air. Rat pituitary cells (#R1220) were purchased from ScienCell Research Laboratories, Inc., (Excel Biomedical, Tainan, Taiwan). R1220 cells reported to express the receptors of gonadotropin releasing hormone (GnRH) were originally isolated from neonate day-8 CD^®^ rats and cryopreserved in primary cultures with further purification and expansion [67]. They were grown in Epithelial Cell Medium (catalog number: 4101; ScienCell).

The embryonic mouse hippocampal cell line (mHippoE-14, CLU198) was obtained from Cedarlane CELLutions Biosystems, Inc. (Hycell International Co.; Taipei, Taiwan). Cells were grown in Dulbecco’s modified Eagle’s medium supplemented with 10% fetal bovine serum (*v/v* percent) and 2 mM L-glutamine. Culture medium was changed every two or three days, and cells underwent passed when they reached confluence. The experiments were performed 5 or 6 days after cells had been cultured (60–80% confluence).

### 4.3. Electrophysiological Measurements

Shortly before the experiments, we dissociated cells (i.e., GH_3_, R1220 or mHippoE-14 cells) and transferred an aliquot of cell suspension to a custom-made recording chamber mounted on the stage of DM-IL inverted fluorescence microscope (Leica; Major Instruments, Kaohsiung, Taiwan). We bathed cells at room temperature (20–25 °C) in HEPES-buffered normal Tyrode’s solution, the composition of which is stated above. The electrodes were prepared from Kimax-51 capillaries (#34500 [1.5–18 mm in outer diameter]; Kimble, Dogger, New Taipei City, Taiwan) by either a P-97 Flaming/Brown puller (Sutter, Novato, CA, USA) or a Narishige PP-830 puller (Narishige; Major Instruments, New Taipei City, Taiwan), and we then fire-polished their tips with MF-83 microforge (Narishige). As filled with different internal solutions, their resistances ranged from 3 to 5 MΩ. Recordings of membrane potential or ionic currents were measured in the whole-cell or cell-attached configuration of the standard patch-clamp technique with an RK-400 patch amplifier (Bio-Logic, Claix, France) [68]. The liquid junction potentials were zeroed shortly before giga-seal formation was made, and the whole-cell data were corrected.

### 4.4. Data Recordings

The signals containing both potential and current traces were stored online in an Acer SPIN-5 touchscreen display computer (SP513-52N-55WE; Taipei, Taiwan) at 10 kHz connected to Digidata 1440A interface (Molecular Devices; Bestogen Biotech, New Taipei City, Taiwan), which was used for efficient analog-to-digital/digital-to-analog conversion. During the recordings, the latter device was operated by pCLAMP 10.7 software (Molecular Devices) run under Windows 10 (Redmond, WA, USA), and the signals were simultaneously displayed on an LCD monitor through USB type-C connection. Current signals were low-pass filtered at 2 kHz with FL-4 four-pole Bessel filter (Dagan, Minneapolis, MN, USA) to minimize background noise. As high-frequency stimuli were necessarily applied, an Astro-med Grass S88X pulse stimulator (Grass, West Warwick, RI, USA) was employed. After the data were digitally collected, we off-line analyzed them using various analytical tools that include LabChart 7.0 program (ADInstruments; Gerin, Tainan, Taiwan), OriginPro 2016 (OriginLab; Schmidt Scientific, Kaohsiung, Taiwan) and custom-created macros run under Microsoft Excel^®^ 2016 (Redmond, WA, USA).

### 4.5. Data Analyses

To determine the percentage increase of KYNA or KYNA-A4 on *I*_K(M)_, current amplitude at the concentration of 1 mM KYNA or KYNA-A4 taken as 100%, and those during exposure to varying KYNA or KYNA-M1 concentrations (1 μM–1 mM) were analyzed and compared. To measure *I*_K(M)_, we kept cells bathed in high-K^+^, Ca^2+^-free solution, and the depolarizing voltage command from −50 to −10 mV was applied. The *I*_K(M)_ amplitude during the application of KYNA or KYNA-A4 were compared with those measured after subsequent addition of linopirdine (10 μM). The concentration-response data for stimulation of *I*_K(M)_ were thereafter least-squares fitted to the modified Hill equation (i.e., multi-parameter logistic equation). That is,

(1) $$\mathrm{Percentage}\;\mathrm{increase}\;\left(\%\right)=\frac{{\left[C\right]}^{n_{H}}\times E_{max}}{{\left[C\right]}^{n_{H}}+EC_{50}^{n_{H}}}$$

where [*C*] represents the KYNA or KYNA-A4 concentration applied; *E_max_* is the maximal stimulation of *I*_K(M)_ (i.e., linopirdine-sensitive current) caused by KYNA or KYNA-A4; and *n_H_* or EC_50_ is the Hill coefficient or the concentration of KYNA or KYNA-A4 required for 50% stimulation, respectively.

The relationship between the membrane potentials and the *I*_K(M)_ conductance obtained with and without the application of KYNA (30 μM) or KYNA-A4 (10 μM) was appropriately fitted with a modified Boltzmann function (or the Fermi-Dirac distribution) of the following form [37]:

(2) $$G=\frac{G_{max}}{1+e^{\left[\frac{-\left(V-V_{1/2}\right)qF}{RT}\right]}}$$

where *G_max_* denotes the maximal conductance of *I*_K(M)_, *V*_1/2_ is the voltage at which half-maximal activation of the current is achieved, *q* is the apparent gating charge, *F* is Faraday’s constant, *R* is the universal gas constant, and *T* is the absolute temperature. 

### 4.6. Analyses of Single M-Type K^+^ (K_M_) Channels

Single K_M_-channel currents recorded from GH_3_ cells were analysis using pCLAMP 10.7 program. We evaluated single-channel amplitude obtained with or with the addition of KYNA or KYNA-M1 by fitting Gaussian distributions to the amplitude histograms of the closed (resting) or open state. The probabilities of K_M_ channel that would be open were defined *N*·*P*_O_, which was estimated using the following expression:

(3) $$N\times P_{O}=\frac{A_{1}+2A_{2}+3A_{3}+4A_{4}+\ldots +nA_{n}}{A_{0}+A_{1}+A_{2}+A_{3}+\ldots A_{n}}$$

where *N* is the number of active K_M_-channels in the patch examined, *A*_0_ the area under the curve of an all points histogram corresponding to the closed (resting) state, and *A*_1_…..*A_n_* the histogram area that indicate the level of distinct open state for 1 to *n* channels in the patch. The single-channel conductance of K_M_ channels was calculated using a linear regression with mean values of single-channel amplitudes measured at different level of membrane potentials relative to the bath.

### 4.7. Statistical Analyses

Linear or nonlinear (e.g., Hill or Boltzmann equation and single exponential) curves fitting to data sets demonstrated here were performed from the goodness-of-fit test using either Microsoft Excel 2016 (Redmond, WA, USA) or 64-bit OriginPro 2016 (OrignLab). The values are provided as means ± standard error of mean (SEM) with sample sizes (*n*), which indicate the number of cells collected. The Student’s *t*-test (paired or unpaired) or one-way analysis of variance followed by post-hoc Fisher’s least-significance difference test for multiple-range comparisons, were implemented for the statistical evaluation of difference among means. Probability with *p* < 0.05 was considered statistically significant, unless noted otherwise.

## Data Availability

Data available in a publicly accessible repository.

## Abbreviations

AP	Action potential
EC_50_	The concentration required for 50% stimulation
HCN channel	Hyperpolarization-activated cyclic nucleotide-gated ion channel
*I*_h_	Hyperpolarization-activated cation current
*I*_K(DR)_	Delayed-rectifier K^+^ current
I-V	Current versus voltage
*I*_K(erg)_	*Erg*-mediated K^+^ current
*I*_K(M)_	M-type K^+^ current
*I*_Na_	Voltage-gated Na^+^ current
K_M_ channel	M-type K^+^ channel
KYNA	Kynurenic acid
KYNA-M1	3-(morpholinomethyl)-4-oxo-1,4-dihydroquinoline-2-carboxylic acid
KYNA-A4	*N*-(2-(dimethylamino)ethyl)-3-(morpholinomethyl)-4-oxo-1,4-dihydroquinoline-2-carboxamide
NMDAR	*N*-methyl-D-aspartate receptor
SEM	Standard error of mean
TEA	Tetraethylammonium chloride
TTX	Tetrodotoxin
